# Discovery of Human-Specific Immunodominant *Chlamydia trachomatis* B Cell Epitopes

**DOI:** 10.1128/mSphere.00246-18

**Published:** 2018-08-01

**Authors:** K. Shamsur Rahman, Toni Darville, Ali N. Russell, Catherine M. O’Connell, Harold C. Wiesenfeld, Sharon L. Hillier, Erfan U. Chowdhury, Yen-Chen Juan, Bernhard Kaltenboeck

**Affiliations:** a Department of Pathobiology, College of Veterinary Medicine, Auburn University, Auburn, Alabama, USA; b Department of Pediatrics, University of North Carolina at Chapel Hill, Chapel Hill, North Carolina, USA; c Department of Obstetrics, Gynaecology and Reproductive Sciences, the University of Pittsburgh School of Medicine and the Magee-Womens Research Institute Pittsburgh, Pittsburgh, Pennsylvania, USA; Parasitology Services

**Keywords:** B cell epitopes, *Chlamydia pneumoniae*, *Chlamydia trachomatis*, ELISA, cross-reactivity, diagnosis, microimmunofluorescence, peptide antigens, serology, serovar, species-specific

## Abstract

*Chlamydia* species-specific serology is compromised by cross-reactivity of the gold standard microimmunofluorescence (MIF) or commercial enzyme-linked immunosorbent assays (ELISAs). This study was conducted to discover novel C. trachomatis-specific peptide antigens that were recognized only by the antibody response of the natural human host. We evaluated a library of 271 peptide antigens from immunodominant C. trachomatis proteins by reactivity with 125 C. trachomatis antibody-positive sera from women with PCR-confirmed C. trachomatis infection and 17 C. trachomatis antibody-negative sera from low-risk women never diagnosed with C. trachomatis infection. These C. trachomatis peptide antigens had been predicted *in silico* to contain B cell epitopes but had been nonreactive with mouse hyperimmune sera against C. trachomatis. We discovered 38 novel human host-dependent antigens from 20 immunodominant C. trachomatis proteins (PmpD, IncE, IncG, CT529, CT618, CT442, TarP, CT143, CT813, CT795, CT223, PmpC, CT875, CT579, LcrE, IncA, CT226, CT694, Hsp60, and pGP3). Using these human sera, we also confirmed 10 C. trachomatis B cell epitopes from 6 immunodominant C. trachomatis proteins (OmpA, PmpD, IncE, IncG, CT529, and CT618) as host species-independent epitopes that had been previously identified by their reactivity with mouse hyperimmune sera against C. trachomatis. ELISA reactivities against these peptides correlated strongly with the C. trachomatis microimmunofluorescence (MIF) text results (Pearson’s correlation coefficient [*R*] = 0.80; *P* < 10^−6^). These C. trachomatis peptide antigens do not cross-react with antibodies against other *Chlamydia* species and are therefore suitable for species-specific detection of antibodies against C. trachomatis. This study identified an extended set of peptide antigens for simple C. trachomatis-specific ELISA serology.

**IMPORTANCE** Current serological assays for species-specific detection of anti-*Chlamydia* species antibodies suffer from well-known shortcomings in specificity and ease of use. Due to the high prevalences of both anti-C. trachomatis and anti-C. pneumoniae antibodies in human populations, species-specific serology is unreliable. Therefore, novel specific and simple assays for chlamydial serology are urgently needed. Conventional antigens are problematic due to extensive cross-reactivity within *Chlamydia* spp. Using accurate B cell epitope prediction and a robust peptide ELISA methodology developed in our laboratory, we identified immunodominant C. trachomatis B cell epitopes by screening performed with sera from C. trachomatis-infected women. We discovered 38 novel human host-dependent antigens from 20 immunodominant C. trachomatis proteins, in addition to confirming 10 host-independent mouse serum peptide antigens that had been identified previously. This extended set of highly specific C. trachomatis peptide antigens can be used in simple ELISA or multiplexed microarray formats and will provide high specificity and sensitivity to human *C. trachomatis* serodiagnosis.

## Abstract (precis)

Current serological assays for species-specific detection of anti-*Chlamydia* species antibodies suffer from well-known shortcomings in specificity and ease of use. Due to the high prevalences of both anti-C. trachomatis and anti-C. pneumoniae antibodies in human populations, species-specific serology is unreliable. Therefore, novel specific and simple assays for chlamydial serology are urgently needed. Conventional antigens are problematic due to extensive cross-reactivity within *Chlamydia* spp. Using accurate B cell epitope prediction and a robust peptide ELISA methodology developed in our laboratory, we identified immunodominant C. trachomatis B cell epitopes by screening performed with sera from C. trachomatis-infected women. We discovered 38 novel human host-dependent antigens from 20 immunodominant C. trachomatis proteins, in addition to confirming 10 host-independent mouse serum peptide antigens that had been identified previously. This extended set of highly specific C. trachomatis peptide antigens can be used in simple ELISA or multiplexed microarray formats and will provide high specificity and sensitivity to human *C. trachomatis* serodiagnosis.

## INTRODUCTION

Obligate intracellular bacteria of the genus *Chlamydia* infect virtually all vertebrates and cause largely chronic and asymptomatic disease conditions (1, 2). The principal human chlamydial pathogens are C. trachomatis and C. pneumoniae (1, 2). *C. trachomatis* serovars A to C cause ocular infection and are the leading causes of preventable blindness, affecting tens of millions of people in developing countries (3). *C. trachomatis* serovars D to K cause genitourinary tract infections, and serovars L1 to L3 cause lymphogranuloma venereum (1, 3). Infections with C. trachomatis genital serovars remain clinically silent in most men and women, but in women, they can ascend to the upper genital tract, leading to pelvic inflammatory disease, infertility, and ectopic pregnancy (4). The single human serovar of C. pneumoniae is a common cause of respiratory infection; such infections lead to pharyngitis, bronchitis, and community-acquired pneumonia (1, 2, 5) and have been associated with atherosclerosis (1, 2, 6, 7).

The remaining 9 chlamydial species have animal hosts (8, 9). C. psittaci infects birds, and C. abortus causes abortion in ruminants; both occasionally cause severe zoonotic human infection. C. felis transmitted from cats is thought to sporadically cause human follicular conjunctivitis or atypical pneumonitis (9). Other chlamydial species are endemic in swine (C. suis), ruminants (C. pecorum), and poultry (C. gallinacea), with poorly understood public health impact (8, 9). C. caviae, C. avium, and C. muridarum are found in guinea pigs, birds, and rodents, respectively, but their significance with respect to epidemiology and public health is largely unknown (8, 9).

Nucleic acid amplification tests (NAAT) are most commonly used for diagnosis of chlamydial infections and for DNA sequence-based differentiation of chlamydiae (3, 6, 9–12), but they provide information only at a single point in time. In contrast, serological assays (13–20) have the power to indicate the history of exposure to an infectious agent and are generally preferable to antigen detection for epidemiological or retrospective analyses. The microimmunofluorescence (MIF) test for detection of antichlamydial antibodies has remained the gold standard since its introduction (21–26). MIF is performed as an indirect fluorescent antibody technique that enables microscopic observation of captured antibody on fixed whole chlamydial elementary bodies (EBs) (21, 23, 24, 26). This is a painstaking technique that requires extensive technical expertise, imposing a risk of high interlaboratory variation in results. The high prevalence of C. pneumoniae respiratory infection in children (27–29) complicates results of serological studies of C. trachomatis due to the possibility of seropositivity arising from a remote C. pneumoniae infection.

Several studies evaluated the suitability of C. trachomatis- or C. pneumoniae-specific ELISAs for analysis of immunodominant antigens (13–20). The majority of such antigens are highly conserved within the *Chlamydia* genus, and determination of suitable antigens for species-specific and sensitive ELISAs is difficult (13, 16–18). ELISAs based on whole elementary bodies (EBs), lipopolysaccharide (LPS), major outer membrane protein (MOMP), Omp2, or Hsp60 suffer from lack of specificity due to cross-reactivity of ELISA antigens (13, 16–18). The Pgp3 protein expressed by the chlamydial plasmid has been extensively studied as a candidate C. trachomatis-specific antigen (13, 18, 30–34). This plasmid protein offers the advantage that it is rarely found in human C. pneumoniae isolates (35); thus, C. pneumoniae infections would not confound the specificity of human Pgp3 *C. trachomatis* serology. Recently, Horner et al. (34) remedied suboptimal Pgp3 ELISA sensitivity with a double-antigen sandwich C. trachomatis Pgp3 ELISA, a method that is, however, cumbersome and labor intensive. In addition, the Pgp3 protein is present and highly conserved in most other *Chlamydia* spp. infecting animal hosts (35, 36) and thus cannot be used to resolve cross-reactivity concerns after human exposure to these animal chlamydiae, while it also may be absent in certain C. trachomatis strains following the loss of the plasmid (37, 38). Thus, assays that determine antibody responses against a wide spectrum of C. trachomatis-specific antigens are still needed to improve the specificity and sensitivity of *C. trachomatis* serology.

We previously identified *Chlamydia* species-specific immunodominant B cell epitopes using mouse hyperimmune sera generated by three high-dose intranasal inoculations of mice with live chlamydial organisms (39). These B cell epitopes were used as peptide antigens in ELISAs for detection of species-specific anti-*Chlamydia* antibodies. Reactivities of C. pecorum-specific peptide antigens were confirmed with sera from cattle, the natural host of C. pecorum (39). However, reactivity of C. trachomatis and C. pneumoniae peptide antigens has not been confirmed with sera of naturally infected humans.

In the present study, we first expanded our bank of *Chlamydia* species-specific mouse sera to all 11 chlamydial species and used it to identify additional immunodominant B cell epitopes. We then confirmed that the mouse-identified C. trachomatis B cell epitopes (referred to as “host-independent” epitopes) are similarly immunodominant in humans, using sera from young women actively infected with C. trachomatis, many of whom also had documented a prior chlamydial genital infection(s). Since antibody responses can be influenced by host-dependent expression of protein antigens in natural versus natural hosts (40), we also sought to identify “host-dependent” C. trachomatis B cell epitopes that might be immunodominant in human infection but fail to elicit antibodies in the heterologous murine host. Since C. pneumoniae is a common infectious pathogen in children and young adults, we also utilized the human sera to probe for host-independent and -dependent B cell epitopes specific for this respiratory pathogen. Using this approach, we have identified highly species-specific C. trachomatis and C. pneumoniae peptide antigens.

## RESULTS

### Mouse-reactive *Chlamydia* species-specific peptide antigens.

**FIG 1:**
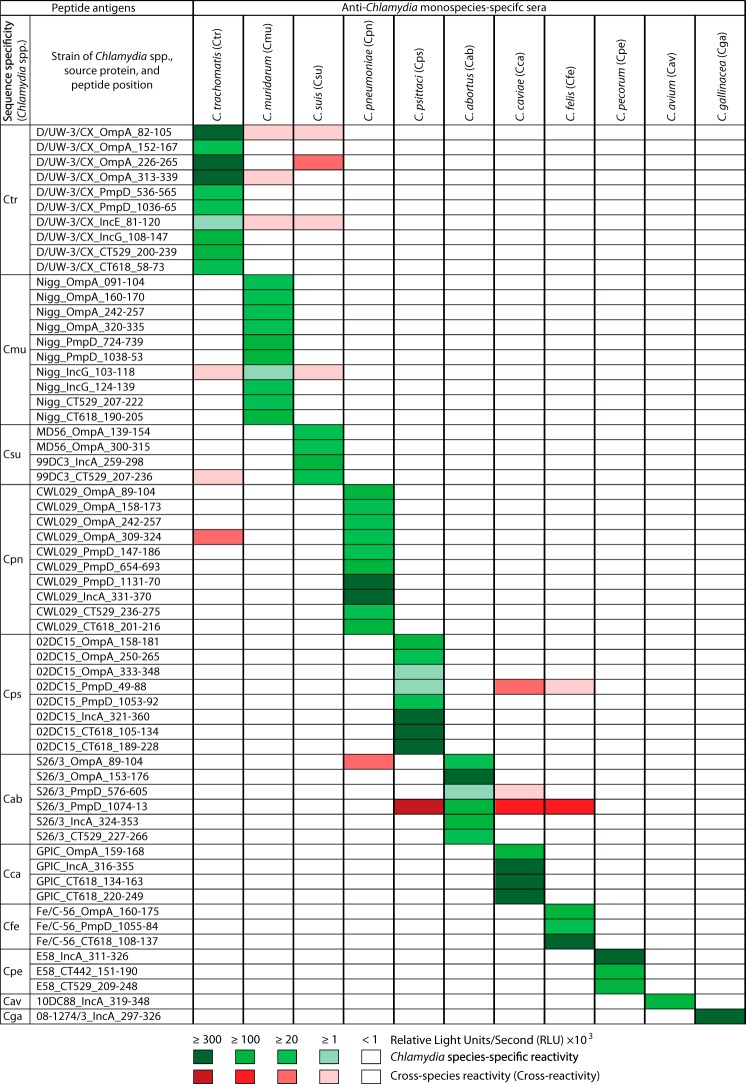
Reactivities of 60 peptide antigens from 11 *Chlamydia* species with *Chlamydia* species-specific mouse sera. Each peptide was ELISA tested with 11 pools of 9 to 50 hyperimmune mouse sera obtained by 3× intranasal inoculation with live inocula of a single chlamydial species (39). Green cells represent the reactivity of peptide antigens with their corresponding homologous antiserum pools. Red cells indicate peptide antigen cross-reactivity with nonhomologous antisera (ELISA signals > background + 2 standard deviations [SD]). Green and red color intensities indicate signal strength, and white cells indicate nonreactivity. Peptide designations consist of three-letter *Chlamydia* species acronyms (defined in the headings of columns 3 to 13) followed by strain, source protein, and the amino acid positions of the peptide in the protein. RLU indicates relative light units per second.

Using monospecies-specific mouse antiserum pools, species-specific reactivities of *Chlamydia* peptide antigens were first confirmed or identified for C. avium and C. gallinacea (Fig. 1 and Table S1). The majority of peptide antigens (49 of 60; Fig. 1 and Table S1) reacted with high specificity only with homologous sera. Only 11 peptides (18%) showed cross-reactivity with 1 or more of the 10 heterologous serum pools, in addition to reacting with the corresponding homologous serum pools (Fig. 1 and Table S1). However, these cross-reactivities were observed among closely related chlamydial species, and the majority of the observed cross-reactivities were weak. These results clearly show that these peptide antigens can be used for *Chlamydia* species-specific detection of anti-*Chlamydia* antibodies.

10.1128/mSphere.00246-18.1 TABLE S1  Peptide sequences of 60 murine immunodominant B-cell epitopes from 11 Chlamydia spp. Download TABLE S1, XLSX file, 0.03 MB. Copyright © 2018 Rahman et al. 2018 Rahman et al. This content is distributed under the terms of the Creative Commons Attribution 4.0 International license.

### Antibodies against *Chlamydia* spp. in women with C. trachomatis infections.

**FIG 2:**
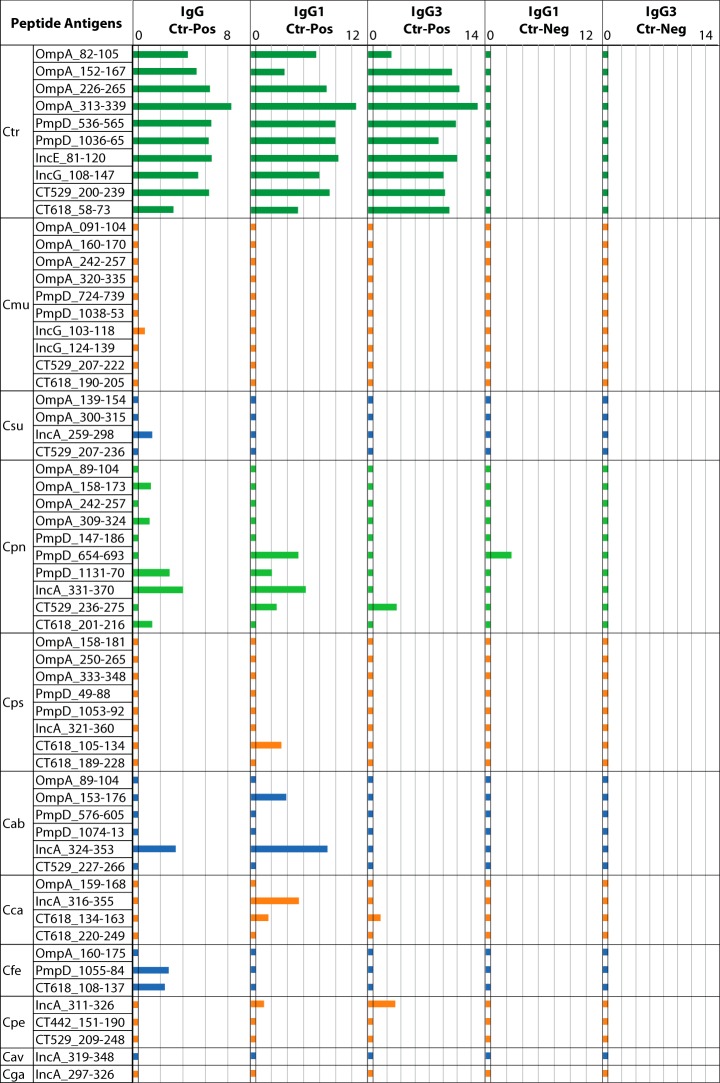
Reactivities of the 60 mouse antiserum-reactive chlamydial peptide antigens with C. trachomatis-positive (Ctr-Pos) and -negative (Ctr-Neg) human serum pools. The reactivity of each of the 60 *Chlamydia* species-specific peptide antigens (Fig. 1) was tested with the human serum pools. The C. trachomatis-positive pool consisted of sera from 125 women with C. trachomatis infection, and the C. trachomatis-negative pool consisted of sera from 17 women never diagnosed with C. trachomatis infection who were EB ELISA negative for anti-C. trachomatis antibodies. Polyclonal anti-human IgG HRP conjugate was used for detection of bound total IgG, and monoclonal antibody conjugates were used to detect bound long-lived IgG1 or short-lived IgG3 isotypes. Peptide reactivities are shown in Log_2_ RLU signal bars. Different colors are used for the chlamydial species for convenient visualization.

To ascertain the suitability of the sera for identification of immunodominant C. trachomatis B cell epitopes, the set of 60 *Chlamydia* species peptide antigens (Fig. 1) was tested with polyclonal anti-IgG and monoclonal anti-IgG1 and anti-IgG3 conjugates for reactivity with the C. trachomatis-positive serum pools (Fig. 2). All 10 C. trachomatis peptides showed consistently strong IgG, IgG1, and IgG3 reactivity with the C. trachomatis-positive pool (Fig. 2). This result confirmed that both long-lived IgG1 and short-lived IgG3 were present in the C. trachomatis-positive serum pool, and this finding was in agreement with the recent C. trachomatis infection history of the study subjects. None of the C. trachomatis peptide antigens showed reactivity with the C. trachomatis-negative serum pool (Fig. 2), confirming the specificity of these antigens. Further evidence for the overall specificity of peptide antigens is the fact that all 10 peptides of C. muridarum, a chlamydial species closely related to C. trachomatis, showed no cross-reactivity with the anti-C. trachomatis sera.

C. pneumoniae peptides from non-OmpA source proteins (PmpD, IncA, CT529, and CT618) showed substantial reactivities with the C. trachomatis-positive serum pools, particularly for long-lived total IgG and IgG1 antibodies (Fig. 2). These data suggest that the study human sera, particularly the sera from 125 women with active C. trachomatis infection, also retained moderate amounts of anti-C. pneumoniae antibodies. Combined with the reactivity of only a single CT529 peptide with short-lived IgG3 antibodies, these results mainly represent past C. pneumoniae infections. Importantly, weak reactivity of only a few peptide antigens of C. abortus, C. felis, C. caviae, C. pecorum, C. pecorum, and C. suis suggests sporadic exposure of human hosts to *Chlamydia* spp. from nonhuman hosts (Fig. 2), given that these peptide antigens showed highly species-specific reactivity with mouse sera (Fig. 1).

### Reactivities of host-independent peptide antigens with anti-C. trachomatis MIF titer-ranked subpools.

**FIG 3:**
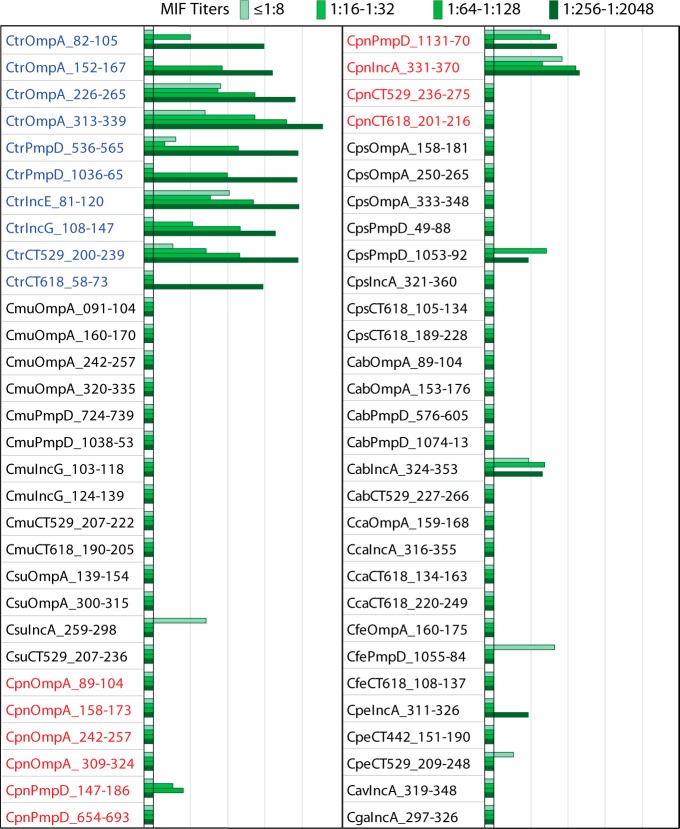
Anti-C. trachomatis MIF titer-dependent IgG reaction intensity of the 60 chlamydial peptide antigens. C. trachomatis-positive sera from 108 women with known anti-C. trachomatis microimmunofluorescence (MIF) test results were combined by MIF titer into 4 subpools of 19 to 35 sera. Reactivities of the 4 subpools, indicated by color intensity, with peptides from all 11 chlamydial species (Fig. 1) are shown in Log_2_ RLU IgG signal bars on the scale as described for Fig. 2. All 10 C. trachomatis peptides showed a signal above background, and the signals correlated highly significantly with MIF titers (*R* = 0.80; *P* < 10^−6^). Three C. pneumoniae peptides and six peptides from other *Chlamydia* species showed a signal above background that did not correlate with MIF titers (*P* = 0.78).

To determine anti-C. trachomatis antibody-dependent signal intensity, the set of 60 mouse-reactive *Chlamydia* species peptides was also tested for reactivity with the four human serum subpools ranked by the C. trachomatis MIF titers of the constituent individual sera (Fig. 3). The reactivities of 10 C. trachomatis peptide antigens correlated strongly with the C. trachomatis MIF titers of 4 C. trachomatis-positive serum subpools (*R* = 0.80; *P* < 10^−6^) (Fig. 3). For instance, the serum subpool with the highest anti-C. trachomatis MIF titers (1:256 to 1:2,048) showed the highest reactivity with all 10 individual C. trachomatis-specific peptides (Fig. 3). Signals above background were observed for 3 C. pneumoniae peptide antigens and for 6 from 5 other chlamydial species (C. abortus, C. felis, C. suis, C. pecorum, and C. psittaci). As expected, these reactivities did not correlate with the anti-C. trachomatis MIF titers of the 4 subpools (*P* = 0.78). Thus, the host-independent peptide antigens of C. trachomatis, but not those of other chlamydial species, showed anti-C. trachomatis MIF titer-dependent reactivity.

Overall, these results confirmed the specific reactivity of *the* C. trachomatis peptide antigens (Fig. 1 to 3). Additionally, the presence of high-titer anti-C. trachomatis antibodies in the serum pools from infected women (Fig. 2 and 3) indicated that these sera were suitable for discovering additional immunodominant C. trachomatis B cell epitopes that had not previously been recognized by mouse hyperimmune sera (39). Antibodies against C. pneumoniae were the second most prevalent antichlamydial antibodies in the human sera (Fig. 2 and 3), in agreement with the high prevalence of C. pneumoniae infections in humans (1, 2, 5–7). This opened the possibility to also identify, by the use of these sera, C. pneumoniae B cell epitopes that were not recognized by anti-C. pneumoniae sera from the heterologous murine host.

### Identification of host-dependent B cell epitopes of C. trachomatis and C. pneumoniae.

**FIG 4:**
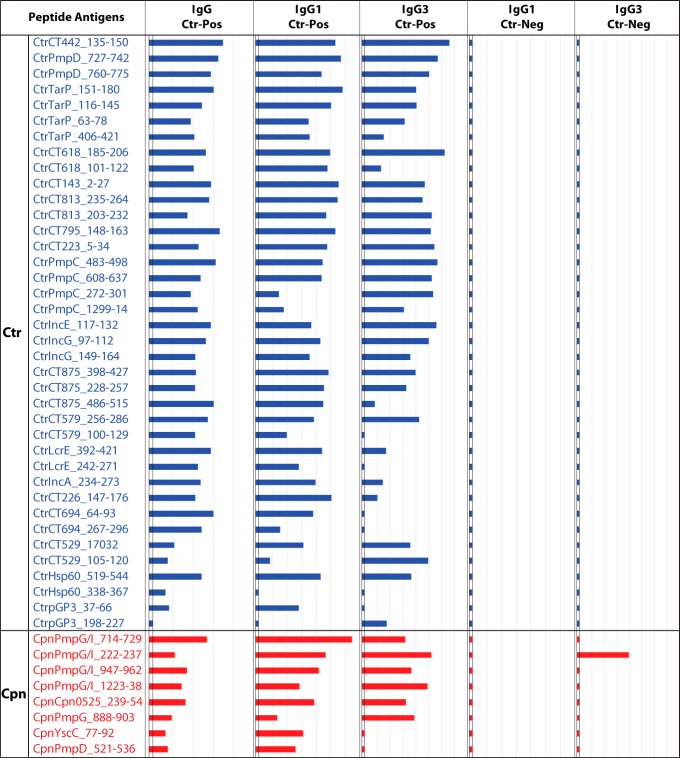
Host-dependent C. trachomatis and C. pneumoniae peptide antigens specifically reactive with human C. trachomatis-positive sera but not with immune mouse sera. A library of 271 C. trachomatis and 153 C. pneumoniae peptide antigens was screened with the C. trachomatis-positive and -negative human serum pools. These peptides had previously been nonreactive in screens performed with hyperimmune monospecies-specific anti-C. trachomatis and anti-C. pneumoniae mouse sera (39). A total of 100 peptides showed reactivity above background, and the top-ranked 38 C. trachomatis and 8 C. pneumoniae peptide antigens derived from immunodominant proteins are shown. Signal intensities are shown in Log_2_ RLU bars as described for Fig. 2.

In the preceding study that identified the set of *Chlamydia* species-specific peptide antigens (39), many more predicted peptides did not react with hyperimmune mouse sera. Therefore, in the present study, we rescreened the C. trachomatis and C. pneumoniae peptides of the library of mouse nonreactive antigens with the human C. trachomatis-positive and -negative serum pools. Of the 271 C. trachomatis and 153 C. pneumoniae peptides tested, the set of 38 reactive C. trachomatis and 8 reactive C. pneumoniae peptides is shown in Fig. 4. These C. trachomatis peptides reacted, almost uniformly, with the C. trachomatis-positive serum pool but not with the C. trachomatis-negative pool, indicating high specificity (Fig. 4). As expected, mouse nonreactive C. pneumoniae peptide antigens also reacted with these human serum pools (Fig. 4).

### Reactivities of host-dependent peptide antigens with anti-C. trachomatis MIF titer-ranked subpools.

**FIG 5:**
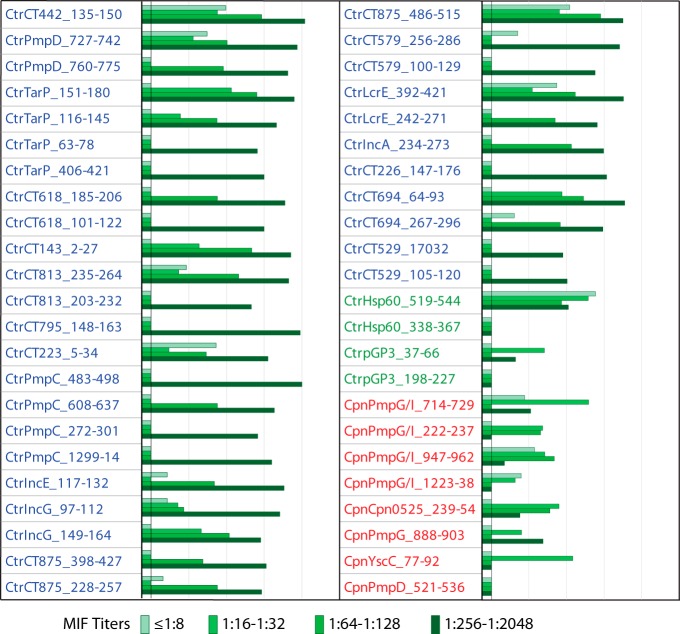
Anti-C. trachomatis MIF titer-dependent IgG reactivity of C. trachomatis but not C. pneumoniae peptide antigens. Thirty-eight C. trachomatis and 8 C. pneumoniae peptide human host-dependent peptide antigens (Fig. 4) were probed with C. trachomatis-positive sera pooled by MIF titer (Fig. 3). Signals are shown in Log_2_ RLU IgG signal bars on the scale as described for Fig. 2. The signals of the C. trachomatis peptides correlated strongly with anti-C. trachomatis MIF titers (*R* = 0.79; *P* < 10^−6^), but those of the C. pneumoniae peptides did not (*P* > 0.61).

To determine the C. trachomatis MIF titer-dependent reactivity of the 46 host-dependent peptide antigens (Fig. 4), they were tested with the four C. trachomatis MIF titer-ranked human serum subpools (Fig. 5). Similarly to the host-independent peptides (Fig. 3), the C. trachomatis peptides, except for the Hsp60 and Pgp3 peptides, reacted most intensely with the subpool with the highest MIF titer (Fig. 5). The reactivities of C. trachomatis peptides also correlated strongly with the MIF titers of the serum subpools (*R* = 0.79; *P* < 10^−6^), except for Hsp60 and Pgp3 peptides. However, the reactivities of C. pneumoniae peptides did not correlate with the C. trachomatis MIF titers (*P* = 0.61). Thus, host-dependent peptide antigens of C. trachomatis, but not of C. pneumoniae, showed anti-C. trachomatis MIF titer-dependent reactivity.

The overall comparison of murine and human host responses to C. trachomatis proteins indicates that a much wider spectrum of proteins is recognized by the antibodies of natural human hosts of C. trachomatis (Fig. 2 to 5: OmpA, PmpD, IncE, IncG, CT529, CT618, CT442, TarP, CT143, CT813, CT795, CT223, PmpC, CT875, CT579, LcrE, IncA, CT226, CT694, Hsp60, and pGP3) than by those of natural murine hosts (Fig. 1: OmpA, PmpD, IncE, IncG, CT529, and CT618). Similarly, humans also produce antibodies against a wider range of C. pneumoniae proteins (Fig. 2 to 5: OmpA, PmpD, IncA, CT529, CT618, PmpG/I, Cpn0525, and YscC) than the natural murine hosts (Fig. 1: OmpA, PmpD, IncA, CT529, and CT618).

### Ranking of candidate C. trachomatis and C. pneumoniae peptide antigens for serological assays.

Table 1 presents the summary of the serological screening results for C. trachomatis and C. pneumoniae peptide antigens that may be suitable for development of species-specific serology. Ten human host-independent and 38 host-dependent peptide antigens of C. trachomatis were ranked by an overall reactivity score (Table 1; see also Table S2 in the supplemental material). Similarly, 10 human host-independent and 8 host-dependent peptide antigens of C. pneumoniae were ranked (Table 1). Only 10 C. trachomatis peptides and 10 C. pneumoniae peptides had been previously identified as B cell epitopes by their reactivity with murine hyperimmune sera (39).

10.1128/mSphere.00246-18.1 TABLE S2  Immunodominant B cell epitopes of C. trachomatis and C. pneumoniae. Download TABLE S2, XLSX file, 0.1 MB. Copyright © 2018 Rahman et al. 2018 Rahman et al. This content is distributed under the terms of the Creative Commons Attribution 4.0 International license.

**TABLE 1:** Immunodominant B cell epitopes of C. trachomatis and C. pneumoniae

Peptide^a^	Sequence^b^	Pooled human sera		Mouse serum status^e^	Peptide sequence % identity		Probability of cross- reactivity (*P*_cross_)	
		Reactivity score^c^	Rank^d^		C. trachomatis strain^f^	C. pneumoniae strain^g^	*C. suis* or C. muridarum^h^	*Chlamydia* spp.^i^
CtrOmpA_313-339	IFDTTTLNPTIAGAGDVKTGAEGQLGD	10.3	1	+	74	<40	0.55	0.01
CtrOmpA_226-265	NVLCNAAEFTINKPKGYVGKEFPLDLTAGTDAATGTKDAS	8.0	5	+	94	55	0.89	0.09
CtrOmpA_152_167	SFNLVGLFGDNENQKT	5.4	27	+	76	50	0.29	0.04
CtrOmpA_82-105	FQMGAKPTTDTGNSAAPSTLTARE	3.9	39	+	64	40	0.06	0.01
CtrCT442_135-150	VVESLSRRNSLVDQTQ	8.9	2	−	99	<40	0.01	0.01
CtrIncE_81-120	LFAISALDVLEDHGLVGCPFKLPCKSSPANEPTVQFFKGK	8.4	3	+	97	<40	0.02	0.01
CtrIncE_117-132	FKGKNGSADKVILVTQ	6.5	17	−	97	<40	0.01	0.01
CtrPmpD_727-742	EKVEEVEPAPEQKDNN	8.2	4	−	100	<40	0.01	0.01
CtrPmpD_536-565	ARAPQALPTQEEFPLFSKKEGRPLSSGYSG	7.7	6	+	100	<40	0.01	0.01
CtrPmpD_1036-65	SGTPVQQGHAISKPEAEIESSSEPEGAHSL	6.8	14	+	98	<40	0.01	0.01
CtrPmpD_760-775	QALFASEDGDLSPESS	6.6	16	−	99	<40	0.01	0.01
CtrTarP_151-180	SSNYDDAAADYEPIRTTENIYESIGGSRTS	7.4	7	−	95	<40	0.01	0.01
CtrTarP_116-145	TSSSDHIPSDYDDVGSNSGDISNNYDDVGS	6.3	21	−	77	<40	0.01	0.01
CtrTarP_63-78	TVVNYTNSASAPNVTV	4.4	33	−	94	<40	0.01	0.01
CtrTarP_406-421	FSKFSGDWDSLVEPMV	3.7	40	−	90	<40	0.07	0.01
CtrCT618_185-206	GNLKQNKPTEGTSKENGFMARL	7.4	8	−	99	<40	0.01	0.01
CtrCT618_58-73	TVSETQQQQLSTIETT	5.0	31	+	100	<40	0.04	0.01
CtrCT618_101-122	KTNPDGSFQLDPVSQQRTLLSP	4.2	37	−	98	<40	0.39	0.01
CtrCT143_2-27	KKPVFTGGAPIPGISTEEGTGVKDQN	7.3	9	−	100	<40	0.20	0.01
CtrCT529_200-239	SAERADCEARCARIAREESLLEVPGEENACEKKVAGEKAK	7.3	10	+	96	<40	0.01	0.01
CtrCT529_17-32	KAFFTQPNNKMARVVN	4.0	38	−	97	44	0.16	0.02
CtrCT529_105-120	SHMKAASQKTQEGDEG	3.4	42	−	96	<40	0.01	0.02
CtrCT813_235-264	AIENLDEMAYEAMEFEKEKHGIKPGRRGSI	7.2	11	−	97	<40	0.01	0.01
CtrCT813_203-232	TVTDLEAAKQQLEEKVTDLESEKQELREEL	6.0	23	−	100	<40	0.01	0.01
CtrCT875_398-427	KGSTHRYAPRDDLSPEGASLAETLARFADD	5.9	24	−	100	<40	0.03	0.01
CtrCT795_148-163	IMDITEIPSINPEFVE	7.0	12	−	99	<40	0.02	0.01
CtrCT223_5-34	ALGTSNGVEANNGINDLSPAPEAKKTGSGL	7.0	13	−	97	<40	0.01	0.01
CtrPmpC_483-498	APSLTEAESDQTDQTE	6.8	15	−	100	<40	0.01	0.01
CtrPmpC_608-637	AIVESTPEAPEEIPPVEGEESTATEDPNSN	6.4	20	−	99	<40	0.01	0.01
CtrPmpC_272-301	ETEQTESNGNQDGSSETEDTQVSESPESTP	4.5	32	−	99	<40	0.01	0.01
CtrPmpC_1299-14	EEQNNNDASNQGESAN	3.7	41	−	98	<40	0.01	0.01
CtrIncG_108-147	RPSDQQESGGRLSEESASPQASPTSSTFGLESALRSIGDS	6.5	18	+	98	<40	0.01	0.01
CtrIncG_97-112	KRSPEEIEGAARPSDQ	6.4	19	−	100	<40	0.02	0.01
CtrIncG_149-164	SGAFDDINKDNSRSRS	5.3	30	−	100	<40	0.01	0.01
CtrCT579_256-286	ALDDVAGTATAVGAKATSGAASAASSAATK	5.6	25	−	100	42	0.52	0.03
CtrCT579_100-129	AQAVHGARDSGFNSDGSATLPSPTGTEVNG	2.1	45	−	100	<40	0.69	0.01
CtrLcrE_392-421	RSSFSSTPPHAPVPQSEIPTSPTSTQPPSP	5.4	26	−	99	<40	0.02	0.04
CtrLcrE_242-271	ATWEDKNHLVPCWDEETKTYNKPLLFIQML	2.9	43	−	100	<40	0.16	0.01
CtrCT875_228-257	NDPLGRRTPNYQSKNPGEYTVGNSMFYDGP	5.4	28	−	100	<40	0.52	0.01
CtrCT875_486-515	QGHYQDPRASDYDLPRASDYDLPRSPYPTP	5.4	29	−	100	<40	0.24	0.02
CtrIncA_234-273	SKTLTSQIALQRKESSDLCSQIRETLSSPRKSASPSTKSS	4.3	34	−	100	<40	0.01	0.01
CtrCT694_64-93	NRGTTTTPSRPVITQANIHHPTISGQGAQP	4.2	36	−	93	<40	0.06	0.01
CtrCT694_267-296	ENEEMNQLILGDQNGQDPQHVQDNSKELQK	2.5	44	−	93	<40	0.05	0.01
CtrCT226_147-176	AQKSKDLELAQKKIEQLQSGLKCVLEESLI	4.2	35	−	97	<40	0.01	0.02
CtrHsp60_519-544	TEALIAEIPEEKPAAAPAMPGAGMDY	6.3	22	−	100	81	0.95	0.74
CtrHsp60_338-367	EKEALEARCESIKKQIEDSSSDYDKEKLQE	0.2	48	−	100	70	0.89	0.69
CtrpGP3_37-66	GTKSTPVAAKMTASDGISLTVSNNSSTNAS	2.0	46	−	96	<40	0.24	0.07
CtrpGP3_198-227	SSGVPNLCSLRTSITNTGLTPTTYSLRVGG	0.9	47	−	97	<40	0.61	0.61
CpnIncA_331-370	QKAESEFIACVRDRTFGRRETPPPTTPVVEGDESQEEDEG	3.5	1	+	<40	95	0.01	0.01
CpnPmpG/I_1223-38	HGQVSYGRNHHNMTTK	4.6	2	−	<40	100	0.01	0.04
CpnPmpG/I_947-62	AGTTLETTTTNNTDGS	5.2	3	−	<40	100	0.01	0.04
CpnPmpG/I_714-729	INNTAKRSGGGIYAPK	6.5	9	−	63	100	0.20	0.29
CpnPmpG/I_222-237	TATDKGGGIYSKEKDS	5.7	14	−	44	98	0.01	0.02
CpnPmpD_1131-70	NKEETLVSAGVQINMSSPTPNKDKAVDTPVLADIISITVD	1.7	4	+	<40	100	0.01	0.01
CpnPmpD_521-536	RSNPKLEQKDSGENIN	1.5	5	−	<40	100	0.01	0.01
CpnPmpD_654-693	EKSLNACSHGDHYPPKTVEEEVPPSLLEEHPVVSSTDIRG	1.3	10	+	<40	100	0.01	0.01
CpnPmpD_147-186	EKISSDTKENRKDLETEDPSKKSGLKEVSSDLPKSPETAV	0.2	12	+	<40	100	0.01	0.01
CpnCT618_201-216	PETISDPENRNKPSAE	0.2	6	+	<40	100	0.01	0.01
CpnOmpA_158-173	FGVKGTTVNANELPNV	0.2	7	+	<40	96	0.01	0.07
CpnOmpA_309-324	AVLNLTAWNPSLLGNA	0.2	15	+	<40	98	0.01	0.64
CpnOmpA_89-104	PTGSAAANYTTAVDRP	0	16	+	<40	98	0.01	0.46
CpnOmpA_242-257	VAFPLPTDAGVATATG	0	17	+	<40	100	0.01	0.16
CpnPmpG_888-903	LLRGSNNYVYNSNCEL	3.3	8	−	<40	100	0.01	0.01
Cpn0525_239-254	AQENSTAKRRRRRAAV	4.8	11	−	63	100	0.29	0.46
CpnYscC_77-92	HTKKTTPGSIPSKVFS	2.1	13	−	<40	100	0.01	0.01
CpnCT529_236-275	RAKESLYNERCALENQQSQLSGDVILSAERALRKEHVATL	1.5	18	+	<40	100	0.01	0.01

a A set of 48 C. trachomatis (Ctr) and 18 C. pneumoniae (Cpn) peptide antigens with highest reactivity is shown. Included are 10 each of previously identified C. trachomatis and C. pneumoniae mouse serum-reactive host-independent peptides (39) and 46 top-ranked host-dependent peptides identified in this investigation by screening 424 mouse nonreactive peptides.

b Only the actual chlamydial sequence of peptide antigens is shown, without the N-terminal biotin or the serine-glycine-serine-glycine spacer that is attached to each peptide (39).

c Reactivity scores are weighted averages of seven values corresponding to the reactivities of total and subpooled sera as described in Materials and Methods.

d The rank of each peptide within the list of 48 C. trachomatis or 18 C. pneumoniae B cell epitopes is shown. C. trachomatis rank is based on reactivity score, and C. pneumoniae rank is based on reaction frequency with 48 individual sera shown in Fig. 6.

e Reactivity (+) or lack of reactivity (−) with C. trachomatis-specific or C. pneumoniae-specific hyperimmune mouse sera is shown (39).

f Average percent amino acid sequence identity with 22 strains representing all major clades of C. trachomatis (Table S2) is shown. Sequences with identities below 40% typically cannot be aligned correctly, and the probability of peptide cross-reactivity is less than 1% (39).

g Average percent amino acid sequence identity with 6 major strains of C. pneumoniae (Table S2) is shown.

h Maximum probability of cross-reactivity (39) with C. muridarum- or C. suis-specific sera based on sequence identity (Table S2) is shown.

i Maximum probability of cross-reactivity (39) with sera specific for the remaining 8 *Chlamydia* spp. (Table S2) is shown.

### Sequence conservation and probability of peptide antigen cross-reactivity.

The intended use of these antigens for species-specific detection of antichlamydial antibodies requires maximum divergence from all other chlamydial species but highest sequence conservation within the target species. By these criteria (<50% sequence identity [SeqID], probability of peptide cross-reactivity [*P*_cross_] ≤ 0.04), the vast majority of these peptide antigens were highly specific for C. trachomatis and were conserved within all *C. trachomatis* serovars but not in the remaining *Chlamydia* spp. (Table 1; see also Table S2). The 30 C. trachomatis peptides from the following 16 proteins fit these criteria: CT442, IncE, PmpD, TarP, CT618, CT529, CT813, CT875, CT795, CT223, PmpC, IncG, LcrE, IncA, CT694, and CT226. The exceptions with a high likelihood of cross-reactivity with other chlamydial species were two C. trachomatis Hsp60 peptides (74% to 85% SeqID) and two pGP3 peptides (40% to 80% SeqID). In addition, 9 peptides of 7 C. trachomatis proteins (OmpA, CT618, CT143, CT529, CT579, LcrE, CT875) had *P*_cross_ values of 0.16 to 0.89 only with C. suis or C. muridarum (40% to 93% SeqID), which are closely related to C. trachomatis.

Five B cell epitope regions of the strongly reactive C. trachomatis OmpA (64% to 76% SeqID) and TarP (77% to 90% SeqID) antigens are polymorphic within C. trachomatis. As indicated in Table S2, the OmpA peptides (variable domain 1 [VD1] to VD4) showed high levels of sequence polymorphism within 22 strains that represent the major clades of C. trachomatis. The highest levels of sequence polymorphisms (Table 1; see also Table S2) were observed within the VD1 region, with only 64% average sequence identity across these 22 C. trachomatis strains (average *P*_cross_ value, ≤0.18), followed by 74% identity for VD4 (*P*_cross_ ≤ 0.42), 76% for VD2 (*P*_cross_ ≤ 0.48), and 94% for VD3 (*P*_cross_ ≤ 0.89). Therefore, these reactive regions with high levels of sequence polymorphism have the potential for use in the design of peptide antigens that discriminate among antibodies against different *C. trachomatis* serovariants.

In contrast, the 13 C. pneumoniae peptides of the following 9 proteins were conserved within C. pneumoniae strains but highly divergent from those of other chlamydial species: IncA, PmpD, PmpG/I, CT618, PmpG, OmpA, Cpn0525, YscC, and CT529 (Table 1; see also Table S2). The exceptions with a high likelihood of reactivity with other chlamydial species were 5 peptides from C. pneumoniae proteins PmpG/I (40% to 69% SeqID), OmpA (40% to 81% SeqID), and Cpn0525 (40% to 75% SeqID).

### Reactivities of C. trachomatis- and C. pneumoniae-specific peptides with individual sera.

To further characterize peptide antigens by testing with individual rather than pooled sera, the most highly ranked 38 C. trachomatis and 18 C. pneumoniae peptides (Table 1) were tested with a panel of 48 individual serum samples from women with C. trachomatis infection (Fig. 6). Results showed that the newly identified host-dependent epitopes had reaction strengths and frequencies similar to those seen with the host-independent epitopes identified previously with mouse sera (Fig. 6; see also Table 1).

**FIG 6:**
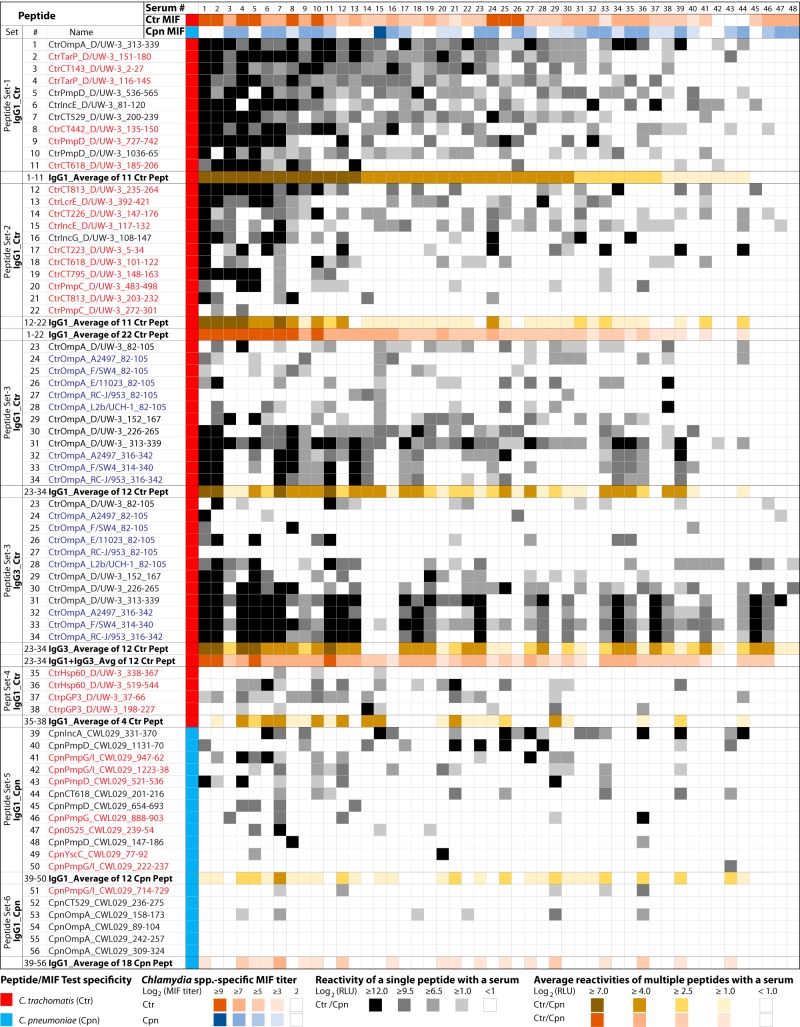
IgG1 and IgG3 antibodies against C. trachomatis and C. pneumoniae peptides in 48 individual sera. Sera from 48 C. trachomatis-positive women that covered the spectrum of C. trachomatis and C. pneumoniae MIF titers (rows 2 and 3), as well as the racial origin of the study subject, were selected. A total of 56 unique C. trachomatis and C. pneumoniae peptide antigens with potential utility for human population surveys were tested with these individual sera. These peptides are divided into 6 sets based on C. trachomatis and C. pneumoniae species, serovar specificity, source proteins, reaction frequency, and the immunoglobulin subclasses detected. The host-independent peptides shown in black font had previously reacted with mouse anti-C. trachomatis sera (39), while the host-dependent peptides shown in red font were not recognized by mouse sera but showed reactivity in this study with human sera. Blue font indicates eight non-serovar D peptides from OmpA variable domains 1 and 2. The column following the peptide designations shows C. trachomatis or C. pneumoniae specificity. Individual sera are numerically identified in the top row and are arranged in order of their average level of IgG1 reactivity with the first set of 11 C. trachomatis peptides. Peptide reactivities with the individual 48 sera are indicated by color intensities (legend at bottom), and absence of reactivity is shown by white cells.

### Correlation of C. trachomatis and C. pneumoniae peptide reactivities and species-specific MIF titers.

Reactivity of the 48 individual sera against C. trachomatis peptide antigens (peptide sets 1 to 3 in Fig. 6) correlated significantly with the anti-C. trachomatis MIF titers of the individual sera (*R* = 0.56 to 0.33; *P* = 10^−4^ to 0.02; Table 2). However, the reactivities of C. trachomatis peptide set 4 (Hsp60, pGP3; Fig. 6) did not correlate with anti-C. trachomatis MIF titers (*P* = 0.42; Table 2). As expected, the signal intensities of all of the sets of C. trachomatis peptides (sets 1 to 4) failed to correlate with anti-C. pneumoniae MIF titers. Similarly, signals of C. pneumoniae peptides (set 5) did not correlate with anti-C. trachomatis MIF titers (*P* = 0.66). Surprisingly, the reactivities of C. pneumoniae peptides (set 5) did not correlate significantly with anti-C. pneumoniae MIF titers (*P* = 0.39). This may have been due to strong human serum reactivities with the non-OmpA proteins of C. pneumoniae but weak reactivities with OmpA antigens (major component of MIF antigens). Overall, the results in Table 2 indicate that C. trachomatis, but not C. pneumoniae, peptide reactivities correlated with the respective MIF titers (Fig. 6). As expected, the ELISA signal intensities of the C. trachomatis peptides in set 1 (Fig. 6) showed strong correlation with other C. trachomatis peptides (set 2) (*R* = 0.81; *P* = 10^−6^; Table 2). In contrast, the reactivities of the C. pneumoniae peptides (set 5) showed only weak correlation with the C. trachomatis peptides in set 1 (*R* = 0.42; *P* = 0.003) (Table 2).

**TABLE 2:** Correlation of C. trachomatis and C. pneumoniae peptide reactivities with species-specific MIF titers

Peptide set^a^	*Chlamydia* species specificity	Source proteins	Antibody isotype	Correlation to^b^:					
				C. trachomatis MIF titer		C. pneumoniae MIF titer		Peptide set 1	
				*P*	*R*	*P*	*R*	*P*	*R*
1 (peptides 1–11)	C. trachomatis	OmpA, TarP, CT143, PmpD. IncE, CT529, CT442, CT618	IgG1	10^−4^	0.56	0.91	–	–	–
2 (peptides 12–22)	C. trachomatis	CT813, LcrE, CT226, IncE, IncG, CT223, CT618, CT795, PmpC	IgG1	10^−3^	0.47	0.20	–	<10^−6^	0.81
3 (peptides 23–34)	C. trachomatis	OmpA, variable domains (VD) 1, 2, 3, 4	IgG1	0.02	0.33	0.32	–	10^−5^	0.65
3 (peptides 23–34)	C. trachomatis	OmpA (VD1 to VD4)	IgG3	0.01	0.36	0.12	–	10^−5^	0.60
4 (peptides 35–38)	C. trachomatis	Hsp60, pGP3	IgG1	0.42	–	0.92	–	0.002	0.44
5 (peptides 39–50)	C. pneumoniae	IncA, PmpD, PmpG/I, CT618, PmpG, Cpn0525, YscC	IgG1	0.66	–	0.39	–	0.003	0.42

a Peptide sets and peptide numbers are identical to those of the peptides shown in Fig. 6.

b Pearson’s correlation coefficient (*R*) and *P* values were calculated in linear regression analyses. The correlations are based on the mean reactivity of a peptide set with each of 48 individual sera (Fig. 6). –, absence of significant correlation or self-comparison.

### Reactivities of serovariant peptides from C. trachomatis OmpA variable domain 1 (VD1) to VD4.

As shown in Fig. 6, the OmpA VD1 peptide of C. trachomatis serovar D/UW-3/CX showed IgG1 reactivity with about 44% of the individual sera (21 of 48 sera) and the VD2, VD3, and VD4 peptides with 38%, 50%, and 79% of the sera, respectively. Testing of 5 additional serovariant peptides from OmpA VD1 increased the frequency of individual serum IgG1 reactivity by 20% (65% with six OmpA VD1 serovariant peptides compared to 44% with a single serovar D VD1 peptide). The use of 3 additional serovariant peptides from OmpA VD4 increased the reactivity frequency by only 4% (83% for four VD4 serovariant peptides compared to 79% with one serovar D VD4 peptide). Reactivities of short-lived IgG3 were almost identical to those of long-lived IgG1, except for distinct differences in some individuals (Fig. 6). These exceptions suggest a potential to distinguish current/recent infections indicated by the presence of IgG3 antibodies from past infections indicated by the presence of IgG1 antibodies. Interestingly, the six serovariant peptides from C. trachomatis OmpA VD1 generated infrequent but distinct signals (Fig. 6). In contrast, the OmpA VD4 peptides showed frequent and common reactivities. Combined with sequence polymorphism data (Table 1; see also Table S2), this result suggests that the use of several serovariant peptides from the highly polymorphic OmpA VD1 region may identify C. trachomatis serovar-specific antibodies and that assaying just a few variant peptides from OmpA VD3 and VD4 may allow detection of all anti-C. trachomatis antibodies.

### C. pneumoniae OmpA and non-OmpA peptide reactivities.

In contrast to the highly reactive C. trachomatis OmpA peptide antigens (Fig. 6), C. pneumoniae OmpA peptides 53 to 56 showed rare and weak reactivity that was much weaker and less frequent than the reactivity seen with dominant peptides 39 to 47 from other C. pneumoniae proteins. All four OmpA variable-domain peptides and the non-OmpA peptides (IncA, PmpD, PmpG/I, CT618, PmpG, Cpn0525, and YscC) (Fig. 6) were highly conserved across all strains of C. pneumoniae (95% to 100% sequence identity) (*P*_cross_, ≤0.91 to 0.95) (Table 1; see also Table S2). Several of those C. pneumoniae non-OmpA peptides (IncA, PmpD, PmpG/I, and CT618; Fig. 6) showed frequent detection of anti-C. pneumoniae antibodies.

## DISCUSSION

In this study, we first reconfirmed the specificities of 60 peptide antigens from the 11 *Chlamydia* spp. (39), using *Chlamydia* monospecies-specific hyperimmune mouse sera (Fig. 1 and Table S1). Subsequent testing of these peptides with the human C. trachomatis-positive and -negative sera confirmed the C. trachomatis-specific reactivity of these peptides with human sera and established the mouse/human host-independent nature of the chlamydial B cell epitopes (Fig. 1, 2, and 3). Next, by screening large numbers of additional C. trachomatis candidate peptide antigens with human sera (Table 1), we identified novel human host-dependent B cell epitope peptide antigens of C. trachomatis that had not previously reacted with mouse anti-C. trachomatis hyperimmune sera (Fig. 4 and 5). Additionally, using the previously identified mouse species-specific peptide antigens (39), we confirmed the presence of both anti-C. pneumoniae and anti-C. trachomatis antibodies in these human sera (Fig. 6). Again, by screening a large number of additional C. pneumoniae candidate peptide antigens with the human sera (Table 1), we identified novel human host-dependent C. pneumoniae B cell epitope peptide antigens (Fig. 4 and 5).

These experiments conclusively established the concept of human host-dependent antibody responses against certain immunodominant B cell epitopes of C. trachomatis and C. pneumoniae. Overall, the identification of many human host-dependent B cell epitopes from two natural human chlamydial pathogens confirmed the concept that the range of antibody responses to chlamydiae in the natural host species is wider than in the experimental murine host (40).

Compared to the frequent reactivities of C. trachomatis and C. pneumoniae peptide antigens (Fig. 2 to 5), only few antigens from C. abortus, C. felis, C. pecorum, and C. psittaci produced ELISA signals, and the signals that those few produced were very weak (Fig. 2 and 3), suggesting sporadic exposure of the human hosts to those animal *Chlamydia* spp. Additionally, all 10 peptide antigens from C. muridarum were nonreactive with the study sera (Fig. 2 and 3), despite the high anti-C. trachomatis antibody levels in the C. trachomatis-positive serum pool. This indicates that these C. muridarum peptide antigens do not cross-react with anti-C. trachomatis antibodies, despite minimal evolutionary divergence between these two chlamydial species. Similarly, reactivity of peptides of C. suis, another species closely related to C. trachomatis, was essentially absent compared to that seen with C. trachomatis (Fig. 2 and 3). These results clearly indicate that these peptides are highly species-specific antigens (Fig. 2).

With the completion of this study, we report now an expanded set of peptide antigens that may be valuable in serodiagnosis of human chlamydial infections (Table 1). These peptide antigens are sufficiently divergent to eliminate virtually any cross-reactivity among antibodies against other *Chlamydia* spp. (see Table S2 in the supplemental material). Derived from the most strongly reactive regions of immunodominant proteins identified in the chlamydial proteome (40–43), they produce very strong ELISA signals under conditions of detection with anti-human antibody conjugates (Fig. 6). The distinguishing characteristic of these peptide antigens is that they react highly specifically with their cognate antibodies and can therefore be used in assays for species- and even serovar-specific detection of antibodies against C. trachomatis and C. pneumoniae. This fills a distinctive void among *Chlamydia* antibody assays for effective species/type-specific detection of antichlamydial antibodies (13–20, 44–48).

A clear absence of reactivity to certain peptides in individual serum samples (“hole in antibody repertoire”), but strong reactivity to others (Fig. 6), points to the molecular uncertainty principle of the antibody response. We attribute this to the stochastic “roll of the dice” nature of immunoglobulin gene recombination (49), which may have failed to create even a single B cell receptor molecule that bound to a B cell epitope, preventing epitope-specific B cell expansion and antibody affinity maturation. These data strongly argue that combined results from multiple peptide antigens, preferably from several proteins, will be required for accurate anti-C. trachomatis antibody detection that approximates the quantitative accuracy of methods using complex multiepitope antigens.

C. trachomatis-seropositive individuals show holes in the antibody repertoire not only against individual peptide antigens but also against whole-protein antigens (40–43, 50–53). Thus, the molecular uncertainty principle of the antibody response applies also to proteins. The use of single antigens in serology therefore inherently compromises sensitivity as well as quantitative accuracy. These disadvantageous performance characteristics of the use of single protein or peptide antigens may inherently impede their suggested use in programs for control and monitoring for trachoma, sexually transmitted infections (STI), or community-acquired pneumonia (52–56).

Interestingly, C. pneumoniae OmpA peptides were strongly reactive with anti-C. pneumoniae mouse sera (Fig. 1), but not with human sera (Fig. 6), while C. trachomatis OmpA peptides reacted strongly with anti-C. trachomatis mouse sera as well as human sera (Fig. 1 and 6). In agreement with these findings, Campbell et al. reported that rabbit anti-C. pneumoniae immune sera recognized C. pneumoniae OmpA poorly but that C. trachomatis immune sera dominantly recognized the respective OmpA proteins (57). These results may explain the finding that anti-C. trachomatis but not anti-C. pneumoniae MIF titers correlated significantly with the corresponding species-specific peptide reactivities (Table 2), since OmpA is the dominant MIF antigen. In agreement with the previous findings (29, 57), these results suggest that the C. trachomatis but not the C. pneumoniae OmpA peptides would be suitable for sensitive antibody detection in serological assays.

Synthetic OmpA peptides have been tested before for *Chlamydia* species-specific serology, and OmpA peptide-based assays provided specificity but lacked sensitivity (58–61). However, those studies used short, spacerless peptide antigens, resulting in low sensitivity that was most likely due to weak antibody binding to the short peptides (62) and/or to steric hindrance of antibody binding (63). Recently, several groups have attempted to identify non-OmpA peptide antigens for C. trachomatis and *C. pneumoniae* serology and to use levels of antibody against 1 to 3 of these peptides as markers for disease (64, 65). However, those assays had low sensitivity, possibly because the peptides do not represent immunodominant B cell epitopes. Therefore, given that only a fraction of *Chlamydia-*infected individuals respond, those assays are inherently of limited use as disease markers. And even if the epitopes were immunodominant, use of multiple antigens is required for sensitive detection of a wide repertoire of anti-C. trachomatis antibodies, as results determined by us and others (40, 41) indicate (Fig. 6).

These synthetic peptide antigens can be readily commercialized in the standard ELISA format as well as in the microarray format (63, 66, 67), and such assays may become widely available tools for chlamydial serology. In a recent study (66), we detected species- and serovar-specific antibodies against *Chlamydia* spp. by a peptide microarray that used several of the peptide antigens described here (Fig. 1; see also Table 1). With the possibility of spotting 196 to 784 peptides on a single chip (67), such microarrays offer unprecedented opportunities for *Chlamydia* species serology by reducing the need for labor-intensive multipeptide ELISAs. Averaged microarray signals of multiple peptide antigens may reliably quantify the overall antichlamydial antibody response, and specific seroreactivity patterns of individual peptides may be associated with a risk of sequelae following chlamydial infection. In conclusion, these peptide antigens will improve C. trachomatis and *C. pneumoniae* serology and will thereby help to more accurately diagnose infections and sequelae and aid in C. trachomatis surveillance and control programs (30–34, 50, 59, 68).

## MATERIALS AND METHODS

### Mouse sera.

*Chlamydia* monospecies-specific mouse sera for each of the 11 *Chlamydia* spp. were used to confirm the specificity of peptide antigens for detection of species-specific antibody. Nine of such hyperimmune sera had been raised in our previous study (39), but additional C. avium- and C. gallinacea-specific sera were raised in the current study. Briefly, EB suspensions of C. avium strain 10DC88 and C. gallinacea strain 08-1274/3 were intranasally inoculated in A/J mice as described previously (39). Sera of 9 to 50 animals infected with C. avium or C. gallinacea were collected into a monospecies-specific serum pool. All animal experimental protocols were approved by the Institutional Animal Care and Use Committees at Auburn University.

### Human sera.

Sera were collected from 125 women with C. trachomatis infection confirmed by NAAT (69) and from 18 healthy, low-risk women who were never diagnosed with C. trachomatis infection. Chlamydia trachomatis MIF titers (A to I, K, and L1 to L3 antigens) were determined for 108 of the 125 C. trachomatis NAAT-positive women at the University of Washington, and 106 were also tested for C. pneumoniae (TW183 antigen) (21–24, 26). Serum samples from 18 healthy women at low risk for C. trachomatis exposure were tested for antibodies against C. trachomatis by an ELISA using a C. trachomatis EB antigen (GenWay Biotech, Inc., San Diego, CA). Seventeen anti-C. trachomatis EB antibody-negative sera were included in this study as C. trachomatis-negative-control sera, and a single positive serum sample was excluded. These samples from the 17 women at low risk for C. trachomatis infection were not tested by MIF. The chlamydial antibody status of all human sera was also characterized by the reactivity with mouse serum-reactive peptide antigens of all 11 chlamydial species.

Among the 125 C. trachomatis-positive women, 74 were African American, 28 were Caucasian, and 23 were Hispanic, Asian, or mixed race. The 17 C. trachomatis-negative healthy women were all Caucasian but were of similar age to the C. trachomatis-positive serum donors. The study protocol was approved by both the Institutional Review Boards for Human Research of the University of Pittsburgh and the University of North Carolina. All participants provided written informed consent at the time of enrollment.

### Pooling of human sera.

Aliquots of serum from the 125 women diagnosed with C. trachomatis were pooled and are referred to here as the C. trachomatis-positive pooled sera. Serum aliquots from the 17 women never diagnosed with C. trachomatis were similarly pooled and are referred to here as the C. trachomatis-negative pooled sera. The C. trachomatis MIF titer for 108 C. trachomatis-infected women was known at study onset, and these 108 samples were used to prepare four serum subpools ranked by C. trachomatis MIF titer. The serum subpools were prepared by mixing equal proportions of 19, 21, 33, and 35 serum samples from women with C. trachomatis MIF titers of 1:4 to 1:8, 1:16 to 1:32, 1:64 to 1:128, and 1:128 to 1:1,048, respectively. To identify additional peptide antigens that showed host-dependent reactivity with human sera, we used the human serum pool or subpools of 125 C. trachomatis-positive and 17 C. trachomatis-negative women to screen a large panel of 271 C. trachomatis and 153 C. pneumoniae peptides that had been nonreactive with mouse sera.

### Peptide antigens.

The B cell epitope prediction approach has been described in detail in preceding studies (39, 62). In brief, we first identified and ranked 72 immunodominant proteins among all chlamydial proteomes on the basis of published data (40–43). On the basis of alignments of each of these 72 individual proteins, suitable regions for identification of genus-, species-, and serovar-specific epitope candidates were further subjected to *in silico* B cell epitope analyses (62). The majority of the peptide antigens were selected from polymorphic protein regions for species-specific reactivity and smaller numbers from highly polymorphic regions (serovar-specific reactivity) or conserved regions (genus-specific reactivity). Conserved B cell epitopes that corresponded to a given *Chlamydia* species but were divergent from those of other *Chlamydia* spp. were selected for analyses of the species specificity of the peptide antigens. Divergent B cell epitopes within a *Chlamydia* species were selected for serovar-specific reactivity.

Peptide antigens were chemically synthesized with N-terminal biotin followed by a serine-glycine-serine-glycine spacer and captured on streptavidin-coated white microtiter plates (Fisher Scientific, Roskilde, Denmark). For antigen optimization of some B cell epitope regions, they were scanned by the use of 16-to-40-mer peptides overlapping by 4 to 12 amino acids (aa), and peptides of different lengths corresponding to regions showing peak reactivity were tested to maximize reactivity.

### ELISA.

Primary antibodies were detected with horseradish peroxidase (HRP)-conjugated secondary antibodies in chemiluminescent ELISAs. Overall methods were employed as described in detail by Rahman et al. (39) with the following modifications. The wash buffer consisted of 0.15 M NaCl, 20 mM Tris-HCl (pH 7.5), 0.025% Tween 20, and 0.001% benzalkonium chloride. The assay diluent consisted of 0.125 M NaCl, 20 mM Tris-HCl (pH 7.5), 0.025% Tween 20, 2% rabbit serum, 0.2% bovine serum albumin, 0.2% casein, 0.2% polyethylene glycol, and 0.005% benzalkonium chloride. The blocking buffer consisted of 0.125 M NaCl, 20 mM Tris-HCl (pH 7.5), 2% rabbit serum, 0.2% bovine serum albumin, 0.2% casein, 0.2% polyethylene glycol, and 0.005% benzalkonium chloride. A polyclonal rabbit anti-human IgG-h+l cross-adsorbed antibody-HRP conjugate was obtained from Bethyl Laboratories (Montgomery, TX) (catalog no. A80-218P). The following monoclonal mouse anti-human antibody conjugates were purchased from Southern Biotech, Birmingham, AL: IgG1 hinge-HRP (catalog no. 9052-05) and IgG3 hinge-HRP (catalog no. 9210-05).

Chemiluminescent detection was chosen because of the signal linearity over 4 to 5 orders of magnitude, and light emission was measured as relative light units (RLU) per second. Background signals were determined for wells without peptide antigen and wells coated with nonspecific peptide antigens (39). The differences between all background signals and all peptide signals were analyzed by one-tailed paired Student’s *t* test. This analysis established that peptide signal correction by subtraction of the background mean value plus 3 standard deviations allowed unequivocal identification of reactive peptides. The polyclonal signal value was expressed as the base value × 10^3^ for comparative displays, since polyclonal anti-human Ig conjugates produced higher chemiluminescent signals than monoclonal Ig conjugates.

### Confirmation of peptide antigens with monospecies-specific anti-*Chlamydia* mouse serum pools.

A set of previously reported (39) and new peptide antigens from each of the 11 *Chlamydia* spp. was first tested with monospecies-specific anti-*Chlamydia* hyperimmune mouse serum pools. Peptide antigens that reacted with the homologous anti-*Chlamydia* serum pool were further tested with the remaining 10 heterologous species-specific mouse serum pools to determine specificity and cross-reactivity.

### Testing of mouse-reactive peptide antigens of 11 *Chlamydia* spp. with human serum pools.

A set of 60 mouse-reactive peptide antigens of all 11 *Chlamydia* spp. was further tested with the pools of C. trachomatis-positive and -negative human sera by using polyclonal anti-human IgG. For confirmation of the presence of and determination of the levels of bound antibody isotypes, reactive peptide antigens were also tested by use of monoclonal conjugates against long-lived IgG1 and short-lived IgG3 antibodies. To determine anti-C. trachomatis MIF titer-dependent signal intensities, these peptide antigens were further tested with the 4 C. trachomatis MIF titer-dependent human serum subpools by using the human anti-IgG conjugate.

### Testing of mouse reactive C. trachomatis peptide antigens with human serum pools.

A large panel of 271 mouse serum-reactive C. trachomatis peptides, predicted as high-scoring B cell epitopes, was first tested with the human serum pool of C. trachomatis-positive sera by using the anti-human IgG polyclonal conjugate. Reactive peptides were further tested as described above for mouse reactive peptides.

### Ranking of C. trachomatis peptide antigens.

C. trachomatis peptides that reacted with the C. trachomatis-positive serum pool but not with the C. trachomatis-negative pool were ranked by an arbitrary reactivity score corresponding to the C. trachomatis-positive serum pool. These scores were determined by combining 7 weighted signal intensities of serum samples as follows—(i) 1/6 weight of the level of polyclonal anti-IgG conjugate reactivity with the pool of 125 serum samples from C. trachomatis-infected women; (ii) 1/12 weight for each polyclonal anti-IgG conjugate reactivity of the 4 MIF titer subpools; and (iii) 1/4 weight each for monoclonal anti-IgG1 and anti-IgG3 conjugate reactivities with the positive serum pool. This approach balanced all reactivities of serum total and subpools and weighted the data from the polyclonal and monoclonal conjugates equally.

### Reactivity of C. trachomatis peptide antigens with 48 individual human sera.

As a final confirmation of the identifications of the novel peptide antigen reagents, the top-ranked peptide antigens of C. trachomatis were tested for IgG1 and IgG3 reactivity with individual serum samples from 48 C. trachomatis*-*infected women. The serum samples were selected based on their distribution across the complete spectrum of C. trachomatis and C. pneumoniae MIF titers, proportional representation of racial origin of the study subjects, and available volume. All tests were performed by using single peptide antigens per well with a single serum sample and a single conjugate.

### Testing of mouse reactive C. pneumoniae peptide antigens with human sera.

To determine and contrast the reactivities of C. pneumoniae peptide antigens and C. trachomatis peptide reactivities, a panel of 153 C. pneumoniae peptide antigens was similarly tested with both C. trachomatis-positive human serum pools and C. trachomatis-negative pools as described above for the C. trachomatis peptide antigens. All 153 C. pneumoniae peptides were initially ranked by combining 7 weighted signal intensities of serum samples as described for C. trachomatis. A set of 18 top-ranked C. pneumoniae peptides was then tested with a panel of 48 individual sera, and the final rank was determined based on the frequency of positive reactivities with these 48 individual sera.

### Statistical analyses.

All statistical analyses were performed and graphical outputs were generated by the use of Microsoft Excel 2016 or the software package Statistica 7.1 (StatSoft, Tulsa, OK). To determine the relationships between continuous variables, Pearson’s correlation coefficient (*R*) values were calculated in linear and polynomial regression analyses from the square root of *R*^2^. *R* values of 0.01 to 0.30 were considered to represent very weak correlations, 0.30 to 0.50 weak correlations, 0.50 to 0.70 moderately strong correlations, 0.70 to 0.90 strong correlations, and 0.90 to 1.00 very strong correlations.

The probability of peptide cross-reactivity (*P*_cross_) was calculated as follows: *P*_cross_ = *e*^(−9.4153 + 0.123223 × percent sequence identity)^/[1 + *e*^(−9.4153 + 0.123223 × percent sequence identity)^]. At 45%, 60%, 75%, and 90% sequence identity (%SeqID), this translates to *P*_cross_ values of 0.02, 0.12, 0.46, and 0.84, respectively. This probability of cross-reactivity of peptide antigens with antibodies against a heterologous B cell epitope had been described earlier based on a large experimental data set (39). It is derived by logistic regression analysis of the observed cross-reactivity of chlamydial peptides with monospecies-specific serum samples against other chlamydial species and the percentage of sequence identity with the corresponding peptide antigens of the respective other chlamydial species (39).
